# Older adults’ communication with an interactive humanoid robot: Expectations and experiences of older adults in verbal and nonverbal communication with a socially interactive humanoid robot: a mixed methods design in Germany

**DOI:** 10.1007/s00391-023-02268-y

**Published:** 2024-01-05

**Authors:** Nicole Strutz, Luis Perotti, Anika Heimann-Steinert, Robert Klebbe

**Affiliations:** Geriatrics Research Group, Charité—Universitätsmedizin Berlin, corporate member of Freie Universität Berlin, Humboldt-Universität zu Berlin, and Berlin Institute of Health, Germany, Reinickendorfer Straße 61, 13347 Berlin, Germany

**Keywords:** Nonverbal communication, Older adults, Humanoid robotics, Human-robot interaction, Socially interactive robots, Nonverbale Kommunikation, Ältere Menschen, Humanoide Roboter, Mensch-Roboter-Interaktion, Sozial interaktive Roboter

## Abstract

**Background and objective:**

One possible approach to counter singularization and loneliness of older adults is the development and implementation of socially interactive robots. Little is known about the expectations and experiences of older adults with socially interactive humanoid robots.

**Material and methods:**

In a mixed-methods design study, user expectations before interaction and the experience and evaluation of verbal and non-verbal communication after interaction with a robot were assessed. Semi-structured interviews were conducted after the interaction.

**Results:**

The majority of older adults expected verbal communication. After the interaction the evaluation of the quality of verbal communication differed. Participants did not expect any form of nonverbal communication. Nonverbal communication was highlighted as particularly positive. Gestures, facial expressions, and body movements were described as confidence building.

**Conclusion:**

The robot’s ability to communicate nonverbally might positively influence older adults’ experience of communication with the robot. In the development of socially interactive robots non-verbal communication should be given more consideration in order to contribute to successful human-robot interaction.

**Supplementary Information:**

The online version of this article (10.1007/s00391-023-02268-y) contains supplementary material, which is available to authorized users.

## Abstract (de)

**Hintergrund und Fragestellung:**

Ein möglicher Ansatz, um der Singularisierung und Einsamkeit älterer Menschen entgegenzuwirken, ist die Entwicklung und Implementierung sozial interaktiver Roboter. Über die Erwartungen und Erfahrungen älterer Menschen mit humanoiden, sozial interaktiven Robotern ist wenig bekannt.

**Material und Methoden:**

In der vorliegenden Mixed-methods-Design-Studie wurden die Erwartungen der Nutzer vor der Interaktion sowie die Erfahrungen und Bewertungen der verbalen und nonverbalen Kommunikation nach der Interaktion mit einem Roboter untersucht. Dazu wurden halbstrukturierte Interviews nach der Interaktion durchgeführt.

**Ergebnisse:**

Die Mehrheit der älteren Menschen erwartete verbale Kommunikation. Nach der Interaktion unterschieden sich die Bewertungen der Qualität der verbalen Kommunikation. Die Teilnehmer erwarteten keine Form der nonverbalen Kommunikation, diese wurde jedoch als besonders positiv hervorgehoben. Gestik, Mimik und Körperbewegungen wurden als vertrauensbildend beschrieben.

**Diskussion:**

Die Fähigkeit des Roboters, nonverbal zu kommunizieren, könnte die Mensch-Roboter-Interaktion älterer Menschen positiv beeinflussen. Bei der Entwicklung von sozial interaktiven Robotern sollte die nonverbale Kommunikation stärker berücksichtigt werden.

In recent years, demographic changes [13, 21] have led to a tense situation regarding loneliness and singularization of older adults [6]. Loneliness is an increasing threat to older people’s mental health [18, 19]. As the aging of the population will tend to increase in the coming years, alternative approaches and concepts to support older people at risk of loneliness are needed. One potential solution to the implications against isolation and singularization is the development and implementation of social robotic devices in everyday home life.

## Introduction

Social robotic devices that are physically embodied like human-like Nao (SoftBank Robotics, Tokyo, Japan) or pet-like Paro (Intelligent System C. Ltd., Nanto, Tomaya, Japan) are not only designed to perform specific care tasks but also to act as a social companion and as a device for social interaction [1]. These kinds of robots are called socially assistive robots and can be divided into two subgroups according to their operational field: service robots and companion robots. Service robots are developed to carry out daily tasks for the user, which often require some sort of physical interaction with the environment. Companion robots are intended to socially interact with the user to enhance their overall well-being [1]. A variety of studies in a review reported positive effects of the use of companion robots on older adults’ psychological health. The use of this kind of robot has been shown to correlate with a decreased feeling of loneliness [3].

In the development of socially interactive robots for use with older adults, there is increasing focus on usability and acceptability. Walters et al. (2008) showed that a human-like appearance and interaction modalities are crucial for usage and acceptance [20]. The imitation of human interaction (especially nonverbal) is a central challenge for companion robots, which are mainly designed for social interaction through speech [8]. According to Six et al. [16] communication is defined by six determinants among which they name messages and interactivity as key characteristics of communication. Messages and interactivity show through verbal and nonverbal signals of communication. Nonverbal interaction features have a prominent role in human communication [15]. Nonverbal signals are deployed to portray personality traits, convey attitudes, express emotions or modulate a verbal message [2, 5]. Companion robots (especially humanoid companion robots) have a physical entity to which the user applies an expectation of certain social skills [9]. In a study with primary school students it was investigated whether the integration of verbal and nonverbal communication by the robot in social interaction increases user engagement [4]. Although there is a lot of important research in the field of robotics and older adults, specifically the question of how older adults evaluate the verbal and nonverbal communication skills of humanoid robots has not yet been comprehensively answered. Current studies often address the age group up to retirement age and not the group of older adults aged 65 years and older or focus on very specific user scenarios of the age group 65 years and older [11, 12].

In the present study we aim to give insights to older adults’ perceptions of the verbal and nonverbal communicative abilities of the humanoid companion robot Pepper. A second aim is to present the dispreferences/preferences of the participants for verbal and nonverbal communication features, such as gestures or eye movements, for interaction with a socially assistive humanoid robot.

## Material and methods

The robot used in this study is called Pepper and was manufactured by the company SoftBank Robotics (Tokyo, Japan). Pepper is frequently used in the context of studies addressing challenges in human-computer interaction [14]. Besides verbal communication via speech, Pepper can communicate nonverbally by performing gestures with its arms and fingers and by moving its torso and head. In addition, it is equipped with illuminated rings around its eyes and illuminated patches on its shoulders, which indicate attention.

The present study used a mixed methods design. Quantitative questionnaires were used before and after the interaction of older adults with the Pepper robot. Qualitative semi-structured interviews were conducted after interaction with the robot. All interviews were recorded using audio equipment and transcribed based on predefined transcription rules. The interview guide included the following questions:

- What were your impressions of the robot at the beginning and after the interaction?
- What kind of communication with the robot did you expect?
- What would be necessary for you to enjoy talking with the Pepper robot?
- What features or capabilities would the Pepper robot need to have for you or others of your age to be comfortable having a conversation with it?

To answer the research questions discussed in this article, the article focuses exclusively on the qualitative part of the study. Information on the quantitative part can be found in Supplementary Data 1 *Material and Methods *and Supplementary Data 2 *Results*.

The participants were adults aged 65 years and older. They were recruited from a participant database of a geriatrics research group.

Participants’ expectations before the interaction with Pepper, as well as their experience and evaluation of verbal and nonverbal communication after the interaction, were explored through a semi-structured interview.

### Interaction scenario with the robot

**Fig. 1:**
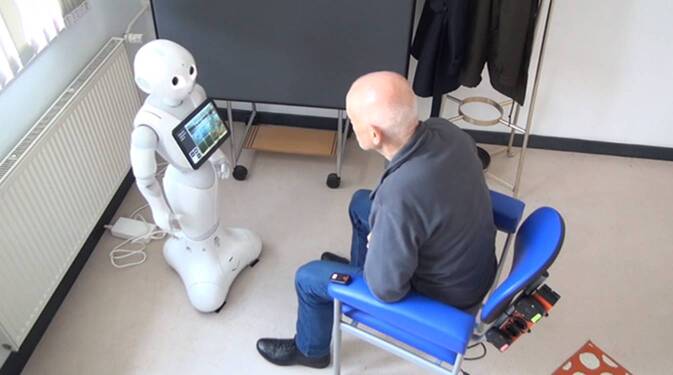
Pepper robot and a study participant during the interaction

The interaction scenario with the robot was structured as follows: the robot stood in the room and was facing the door. The distance between the chair and the robot was designed so that participants could see the robot as a whole (see Fig. 1). Responses could be entered by voice or by tablet.

The participants were proactively greeted by the robot when entering the room. The robot introduced itself, its possibilities for communication and special features by means of verbal and nonverbal communication. The robot indicated that it was only ready to receive verbal information when a stripe on the shoulders was lit up blue.

Participants were asked to play three different interactive games with the robot. These included memory games, guessing names of songs and a virtual walk in the forest, which, among other things required participants to correctly assign tracks to wild animals, twittering to birds and identify berries growing in the forest. It was possible to interact with the robot either by using the touchscreen of the tablet or by voice input.

## Data analysis

### Interviews

The number of participants included in the study was based on the principle of theoretical saturation, which is defined as the point at which “no additional information on the research topic was found whereby the researchers could develop properties of the category” [10]. Field notes were taken for the qualitative analysis and later used in discussions about categories. Based on key aspects of verbal and nonverbal communication such as voice, language and content, gestures, facial expressions and body movements, categories were developed (see Supplementary Data 1). Coding techniques were based on qualitative content analysis.

## Results

### Study population characteristics

The study sample consisted of 21 participants (45% female) with a mean age of 74.6 years (SD =± 5.6 years). Participants generally had a high level of education and were predominantly frequent users of technologies/computers (see Table 1 in Supplementary Data 2). Interviews had a mean length of 16 min 40 s.

### Results of the qualitative study section

From the qualitative interviews, four different themes or categories of expected and experienced interaction were elaborated. These were dialogue, content of communication, use of language and nonverbal characteristics. When looking at the four themes, there were differences in the evaluation of expected and experienced interaction.

### Dialogue

In response to the question “What characteristics or skills would Pepper need to have for other people your age to talk to him?”, one third of the participants (*n* = 7) expected a dialogue with the humanoid robot. As an example, participant #13 said: ‘I really expected that […] there wouldn’t just be a program, but a real dialogue’. When asked, the participant suggested that he had come to this assumption because of a book he had recently read. The remaining 13 participants did not comment on this aspect. About one fifth of the participants (*n* = 4) stated that they had experienced a dialogue. The statements were supplemented by two other arguments: the dialogue was easier than input via touchscreen and it was more fluent than expected. The majority of the participants (*n* = 15) did not experience a dialogue between themselves and the humanoid robot. The participants understood a dialogue as a two-way and sequential conversation, where the robot and human are responding to what the counterpart said. Participant #3 commented: ‘I really thought that he says something and if I also say something, he can then already adjust, not his program, but really a dialogue.’. The statement of participant #9 indicates perceiving the interaction with the robot as a one-way-interaction and not as a dialogue: ‘At some point he says I think I’m talking too much, which is okay, but there’s actually no real dialogue, so to speak.’

### Use of language

#### Before the interaction

A total of 14 statements were made about the conception and expectations of the participants towards the language of the robot. Of these statements seven were made before the interaction, addressing the expectation that the robot would have the ability to interact via speech. It was stated that the expectation came from television/films, books, or previous experience in robotics (‘my expectations were a little too high. Maybe due to the book I have been reading, where the robots could already do everything’; participant #3). Only one statement referred to the fact that language was not expected (participant #13) and two participants (participants #12 and #13) stated that they had not thought about the quality of the robot’s language before participating in the study. Another three participants (participants #3, #15 and #18) expected that the robot would have good-quality language, referring to anticipations of a human, fluid, or ‘normal’ language. While one third of the participants expected spoken language during the interaction, only two participants (participants #19 and #21) commented on what they expected from the robot’s voice before interacting with Pepper. They described the voice as childlike and pleasant.

#### After the interaction

After the interaction with the robot, 17 statements were made, which referred to the evaluation of the experienced speech of the robot. Of the statements 15 included that the participants perceived the robot’s communication as spoken language. Several statements (*n* = 6) referred negatively to the language of the robot. It was mentioned twice that verbal communication with the robot required a certain degree of adaptation to the robot’s language limitations. The time-delayed response of the robot required the subject to wait. This was evaluated negatively. One participant (participant #10) testified that there was no need for the robot to communicate verbally.

#### Nonverbal characteristics

The most important difference between expected and experienced areas of communication was found in nonverbal communication. The participants only made two statements on the complexity of nonverbal communication features such as gestures, mimics, or other bodily expressions. These related to the assumption that the humanoid robot would not move fluidly. After the interaction, the participants made a variety of statements about both gestures and other reactions (which were interpreted as facial expressions), and about the movements experienced (the robot’s body language). The gestures were described with positive statements, such as ‘fluid’, ‘human-like’ and ‘relationship-promoting’, and as ‘creating familiarity’. The eyes of the robot were also described as ‘relationship-promoting’ and ‘familiar’. Furthermore, the ‘blinking’ as feedback and the contact/feedback through the eyes was experienced as ‘positive’. There were three statements (participant #12, #13 and #19) that referred to the movements of the robot, which were experienced as ‘not frightening’ and as ‘amazing’. In addition, the quality of the movements was described as ‘exaggerated’ and ‘awkward’.

#### Statements on potential usage

During the semi-structured interviews, several participants stated that they would not accept the humanoid robot for themselves or their families as a partner. Here some statements (participant #4, #5) referred to a use for adults who are older and/or more frail than they are:‘ I spoke of retirement home.’ and ‘There, I think, so it’s too undemanding for my family.’ (participant #4) and ‘So, my 90-year-old mother-in-law was passionate about playing […]. And he could do that.’ (participant #5).

## Discussion

The participants did not expect a robot with the ability to communicate nonverbally. The reported previous experience of older adults with robots in television, movies, or elsewhere might have affected the expectations towards the abilities of Pepper. This is also reflected by Sundar et al. who stated that past exposure to robots in the media has an impact on older adults’ perceptions of robots [17]. This kind of influence was particularly noticeable in our data when looking at the low number of statements regarding the expectations towards the interactive abilities of the robot. Nevertheless, all older adults in our study engaged in the interaction with the robot and used communication via speech. After the interaction, many positive statements about the communicative abilities of the robot were expressed, which are the core competencies of companion robots [1]. The voice and speech abilities were regarded as surprisingly fluent and advanced. The nonverbal skills were particularly positively highlighted. The gestures, eye blinking, and body movements of the robot were perceived as positive and described as ‘trust-building’ and ‘pleasant’. Although participants did not have any expectations of the nonverbal abilities of the robot, this aspect, which also plays an important role in human-to-human interaction, was a positive surprise for many of the older adults.

Studies have shown that there is a need for robots to act as a partner for social interaction with older adults [1]. In contrast, this need was not expressed by any of the participants in the present study. Our findings concur with those of Frennert et al. [7] who found that older adults tend to evaluate robots as ‘good for others, but not for themselves’. They also stated: ‘Perhaps the unwillingness to imagine having an assistive robot is due to the reluctance to accept the physical and cognitive effects of aging’ [7, p. 26]. In our study, participants could envision robot use for individuals who are more affected by age-related functional limitations than they are.

## Conclusion

The nonverbal communication abilities of the socially interactive robot were highlighted as particularly positive by the older adults. In the development of such robots, the ability to communicate with gestures, body movements, and facial expressions should be given consideration in order to contribute to successful human-robot communication.

## Limitations

It should be noted that the target group of older adults is very diverse and it is difficult to make general statements about their preferences and needs. This diversity is particularly significant regarding the attitude towards technology, their own health status, and the associated need for support as well as the existence of social contacts if attitudes and acceptance towards robots are to be recorded. Highly educated individuals with a high affinity for technology participated in the study. It is possible that our results cannot be generalized to the entire target group of older adults. Furthermore, the communicative capabilities of the robot were limited by restricted internet access due to data protection requirements. This may have influenced the evaluation.

## Conclusion

- Overall, the communicative capabilities of the Pepper robot were rated positively by the older adults.
- The robot’s ability to communicate nonverbally positively influenced older adults’ experience of communication with the robot.
- As described by other studies nonverbal communication was perceived as a crucial factor contributing to successful human-robot interaction. In particular, the voice, facial expressions and gestures were important factors.

### Supplementary Information

Supplementary Data 1— Material and Methods

Supplementary Data 2—Results

## References

[1] Abdi J, Al-Hindawi A, Ng T, Vizcaychipi MP (2018) Scoping review on the use of socially assistive robot technology in elderly care. BMJ Open 8:e18815. 10.1136/bmjopen-2017-018815 29440212 10.1136/bmjopen-2017-018815 PMC5829664

[2] Robin ADAM (1984) Problems of context and criterion in nonverbal communication: a new look at the accuracy issue. Issues Pers. Percept.

[3] Broekens J, Heerink M, Rosendal H (2009) Assistive social robots in elderly care: a review. Gerontechnology 8:94–103. 10.4017/gt.2009.08.02.002.00 10.4017/gt.2009.08.02.002.00

[4] Chang C‑W, Lee J‑H, Chao P‑Y et al (2010) Exploring the possibility of using humanoid robots as instructional tools for teaching a second language in primary school. Educ Technol Soc 13:13–24

[5] DePaulo BM (1992) Nonverbal behavior and self-presentation. Psychol Bull 111:203–243. 10.1037/0033-2909.111.2.203 1557474 10.1037/0033-2909.111.2.203

[6] Fakoya OA, McCorry NK, Donnelly M (2020) Loneliness and social isolation interventions for older adults: a scoping review of reviews. BMC Public Health 20:129. 10.1186/s12889-020-8251-6 32054474 10.1186/s12889-020-8251-6 PMC7020371

[7] Frennert S, Eftring H, Östlund B (2013) What older people expect of robots: a mixed methods approach. In: Herrmann G, Pearson MJ, Lenz A et al (eds) Soc. Robot. Springer International Publishing, Cham, pp S 19–S 29

[8] Han J, Campbell N, Jokinen K, Wilcock G (2012) Investigating the use of non-verbal cues in human-robot interaction with a nao robot. 2012 IEEE 3rd Int Conf Cogn Infocommunications Coginfocom. 10.1109/CogInfoCom.2012.6421937 10.1109/CogInfoCom.2012.6421937

[9] Hegel F, Spexard T, Wrede B et al (2006) Playing a different imitation game: Interaction with an empathic android robot. In: 2006 6th IEEE-RAS Int. Conf. Humanoid Robots. IEEE, University of Genova,, Genova, Italy, pp S 56–61

[10] Hunger I, Müller J (2016) Barney G. Glaser/Anselm L. Strauss: the discovery of grounded theory. Strategies for qualitative research, aldine publishing company: Chicago 1967, 271 S. (dt. Grounded theory. Strategien qualitativer forschung, Bern: Huber 1998, 270 S.). In: Salzborn S (ed) Klass. Sozialwissenschaften 100 Schlüsselwerke Im Portrait. Springer Fachmedien Wiesbaden, Wiesbaden, pp 259–262

[11] Johnson DO, Cuijpers RH (2019) Investigating the effect of a humanoid robot’s head position on imitating human emotions. Int J Soc Robot 11:65–74. 10.1007/s12369-018-0477-4 10.1007/s12369-018-0477-4

[12] Johnson DO, Cuijpers RH, Pollmann K, van de Ven AAJ (2016) Exploring the entertainment value of playing games with a humanoid robot. Int J Soc Robot 8:247–269. 10.1007/s12369-015-0331-x 10.1007/s12369-015-0331-x

[13] Lutz W, Scherbov S (2003) Future demographic change in Europe: the contribution of migration. https://iiasa.dev.local/. Accessed 14 Nov 2023

[14] Pandey AK, Gelin R (2018) A mass-produced sociable humanoid robot: pepper: the first machine of its kind. IEEE Robot Autom Mag 25:40–48. 10.1109/MRA.2018.2833157 10.1109/MRA.2018.2833157

[15] Phutela D (2015) The importance of non-verbal communication. IUP J Soft Ski IJSS 9:

[16] Six U, Gleich U, Gimmler R (2007) Kommunikationspsychologie – Medienpsychologie: Lehrbuch, 1st edn. Beltz PVU, Weinheim Basel

[17] Sundar SS, Waddell TF, Jung EH (2016) The hollywood robot syndrome media effects on older adults’ attitudes toward robots and adoption intentions. In: 2016 11th ACMIEEE Int. Conf. Hum.-Robot Interact. HRI. IEEE, Christchurch, New Zealand, pp S 343–S 350

[18] Tesch-Roemer C, Huxhold O (2019) Social isolation and loneliness in old age. Oxf Res Encycl Psychol. 10.1093/acrefore/9780190236557.013.393 10.1093/acrefore/9780190236557.013.393

[19] Thomas J (2015) Insights into loneliness, older people and well-being. Off Natl Stat

[20] Walters ML, Syrdal DS, Dautenhahn K et al (2008) Avoiding the uncanny valley: robot appearance, personality and consistency of behavior in an attention-seeking home scenario for a robot companion. Auton Robots 24:159–178. 10.1007/s10514-007-9058-3 10.1007/s10514-007-9058-3

[21] (2019) 2019 / [Hrsg.: Statist. Bundesamt, Wiesbaden. Red.-Ltg. Ilka Willand], Redaktionsschluss 1. August 2019. Statistisches Bundesamt, Wiesbaden

