# Supplementary material for: Older adults’ communication with an interactive humanoid robot: Expectations and experiences of older adults in verbal and nonverbal communication with a socially interactive humanoid robot: a mixed methods design in Germany
Source: Z Gerontol Geriatr. 2024 Jan 5;57(5):371–5. doi: 10.1007/s00391-023-02268-y (PMC11315771; doi:10.1007/s00391-023-02268-y)
Supplement: Supplementary file 1 — Supplementary Data 1— Material and Methods [file 391_2023_2268_MOESM1_ESM.docx]

## Methods and Material

## Inclusion criteria

Inclusion criteria were defined as follows: an age of 65 years and older, no previous experience with robots. Exclusion criteria were: the presence of legal care, severe cognitive disorders, psychiatric disorders, severe auditory, motor and visual impairments, current participation in other studies, severe systemic diseases, central nervous system disorders, persistent pain, and cerebrovascular diseases. The inclusion and exclusion criteria were assessed via telephone screening as a self-disclosure.

## Ethical Approval

The study was assessed, positively evaluated and approved by the responsible ethics committee (Ethics Committee of the Charité – Universitätsmedizin Berlin, Number EA2/146/19) prior to commencement. The study was conducted following the guidelines of the World Medical Association according to the Declaration of Helsinki.

## Questionnaires

Statistical analysis was performed using IBM® SPSS® Statistics 25.0 (IBM® Armonk, New York, United States). For the descriptive analysis, mean values and standard deviations were calculated. To test for significant differences between groups, the non-parametric two-sided Wilcoxon signed-rank test was performed. We specified a significance level of 0.05 for the analysis. There were no dropouts or adverse events.

## Qualitative analysis – additional information

The qualitative interview guide was created after consultation with a psychologist and discussion within the research group. It addressed participants’ socio-demographic information and their expectations regarding communication, both with a humanoid robot in general and with the particular robot in the study. Participants were also asked about their impressions at the beginning and end of the communication. They were asked what possible applications they saw for the robot in every-day life and what conditions would have to be met for its use.

The qualitative analysis and data management were supported by Atlas.ti 8.0 scientific software (ATLAS.ti Scientific Software development GmbH, Berlin, Germany). The qualitative content analysis was performed according to Mayring et al. (2010) [15].

In the first step, the categorizing was deductive; based on keywords of communication. These keywords were parts of verbal communication as voice, language and content, and parts of non-verbal communication as gestures, facial expressions, and body movements. In the second step, the examination units were explored iteratively-inductively. To ensure the reliability of the results, we conducted the coding process systematically and transparently.
