# Supplementary material for: Older adults’ communication with an interactive humanoid robot: Expectations and experiences of older adults in verbal and nonverbal communication with a socially interactive humanoid robot: a mixed methods design in Germany
Source: Z Gerontol Geriatr. 2024 Jan 5;57(5):371–5. doi: 10.1007/s00391-023-02268-y (PMC11315771; doi:10.1007/s00391-023-02268-y)
Supplement: Supplementary file 2 — Supplementary Data 2—Results [file 391_2023_2268_MOESM2_ESM.docx]

# Results

## Study Population characteristics

Table 1. Study population characteristics (n=21).

| **Variables** | **n** |
| --- | --- |
| Sex  Female, n (%) | 10 (45) |
| Age; mean (SD) | 74.6 (5.6) |
| Highest level of education (missing=1)  Secondary school certificate  High school graduation  Job training  Graduate degree  PhD or Habilitation | 1  2  6  8  3 |
| Experience with technology, absolute (missing=1)  rarely  occasionally  frequently | 1  1  18 |
| Humanoid Robot ‘*Pepper’* is known  yes | 1 |
